# Differential Impact of an Education-Based Intervention for Patients with Type 2 Diabetes Mellitus in Rural China

**DOI:** 10.3390/ijerph16152676

**Published:** 2019-07-26

**Authors:** Shaofan Chen, Bo Burström, Vibeke Sparring, Dongfu Qian, Kristina Burström

**Affiliations:** 1 Health Outcomes and Economic Evaluation Research Group, Stockholm Centre for Healthcare Ethics, Department of Learning, Informatics, Management and Ethics, Karolinska Institutet, 171-77 Stockholm, Sweden; 2 Equity and Health Policy Research Group, Department of Public Health Services, Karolinska Institutet, 171-77 Stockholm, Sweden; 3 School of Health Policy and Management, Nanjing Medical University, Nanjing 211166, China; 4 Center for Health Policy Studies, Nanjing Medical University, Nanjing 211166, China; 5 Medical Management Centre, Department of Learning, Informatics, Management and Ethics, Karolinska Institutet, 171-77 Stockholm, Sweden

**Keywords:** diabetes care, educational intervention, primary care, rural China

## Abstract

The study aimed to assess the impact of an education-based intervention to improve vertical integration and management of type 2 diabetes mellitus in primary care in rural China. Patients with type 2 diabetes mellitus in three townships in Jingjiang county, Jiangsu Province were randomly divided into intervention and control groups. Participants in the intervention group received an education-based intervention. Patients’ data including the fasting blood glucose (FBG) level, health-related quality of life (HRQoL), and sociodemographic characteristics were collected at baseline (2015) and follow-up (2016). The FBG levels decreased significantly in the intervention group compared to the control group in the overall analysis. In the stratified analysis, FBG levels and some aspects of HRQoL improved in the intervention group more for females, married persons, those with low education, and those in farming or house working. Participants in the control group deteriorated in FBG levels but improved in some aspects of HRQoL. The intervention improved in FBG levels and some aspects of HRQoL among participants. Furthermore, the intervention seemed to differentially benefit females, married persons, lowly educated persons, and those in farming or house working more than other groups. (Trial registration: ISRCTN, ISRCTN13319989. Retrospectively registered 4 April 2017).

## 1. Introduction

Type 2 diabetes mellitus is a major health problem all over the world, including in China where the national prevalence rate of diabetes was 10.9% in 2013 [1]. The prevalence of type 2 diabetes mellitus in rural China increased from 6.1% to 8.2% from 2000 to 2014 [2]. The situation in rural China is difficult, as the number of patients with type 2 diabetes mellitus is increasing faster in rural areas than in urban areas, while the awareness, treatment, and control of diabetes remains lower in rural areas [3,4]. The epidemic peak of type 2 diabetes mellitus is moving from cities to suburbs and rural areas [5]. A large proportion of the poor population live in rural areas, and affordable as well as adequate health care is still not available for rural residents [6].

The previous strategy for diabetes care in rural China focused on the costly hospital care instead of primary care and self-management in rural China [7]. However, appropriate care might not be provided because the government subsidies are still not enough [8]. Although the number of doctors and nurses who work in the primary healthcare institutions are increasing, huge gaps still exist in the licensure and professional education of primary healthcare doctors [9]. A large number of primary care doctors do not have medical certifications [8]. Furthermore, the reimbursement for inpatients is higher than for outpatients, so that patients are more willing to go to hospitals rather than primary care for even minor health issues [10]. Service quality and service capabilities are different at the different levels of health care institutions in rural China [8]. In general, primary care institutions are usually perceived to be the providers of the lowest standard of care [8]. However, the government is trying to shift the diabetes care to primary care institutions and increase healthcare resources to primary care [11].

In response to the urgent need for knowledge and management strategies for both patients with type 2 diabetes mellitus and healthcare professionals in the primary care institutions, education-based interventions supported by experts may be a way to vertically integrate and strengthen diabetes care in rural China. There have been many studies in recent years which focused on educational interventions for diabetes patients in China in order to improve glucose control and empower patients themselves to better manage their disease [12,13]. However, few studies have concentrated on type 2 diabetes mellitus patients in rural areas. Studies focusing on improving blood glucose levels as well as improving certain aspects of health-related quality of life (HRQoL) are also lacking in rural settings. A systematic review showed that behavior change, Body Mass Index (BMI), blood pressure, lipids, and medical costs were not clearly addressed in many studies, and few studies focused on the long-term outcomes and adherence to diabetes education [14]. Meanwhile, education-based interventions tend to be more successful among highly educated people than lowly educated people [15], which is challenging when implementing an intervention in rural areas, where a majority are lowly educated. Due to the obstacles of type 2 diabetes mellitus management in rural China and the lack of studies, more knowledge is needed regarding the feasibility and impact of education-based interventions. Hence, the aim of this study was to assess the impact of an education-based intervention on participants’ health outcomes with a specific focus on how the intervention affected: (a) the fasting blood glucose (FBG) level, (b) HRQoL as measured by EQ-5D-3L, and (c) whether these outcomes differed in different sub groups.

## 2. Materials and Methods 

### 2.1. Study Setting and Intervention

This study is nested within a research project on vertical integration strategies in health services for rural patients with chronic diseases (Studying the Vertical Integration Strategy of Chronic Disease Service Based on Multiple Incentive Mechanisms in Rural China, ISRCTN13319989) [16], which focuses on optimizing the care of patients with type 2 diabetes mellitus and primary hypertension in three pilot counties in rural areas of Jiangsu province. An education-based intervention directed to healthcare professionals and patients was performed to shift the care of patients with type 2 diabetes mellitus and primary hypertension from the hospital to primary care services and to improve vertical integration in healthcare for patients with these conditions. The present study focuses on patients with type 2 diabetes mellitus.

The intervention was conducted from November 2015 to November 2017 by service teams assembled by the local health authorities. Doctors from the county hospital led the service teams. The service teams in the intervention areas consisted of healthcare professionals (doctors, nurses, public health physicians, diabetes specialists) from all three levels of healthcare institutions in rural China: county-level hospitals, township health centers, and village clinics. The healthcare professionals in the intervention townships received professional skills training, regular meetings to discuss team work progress, team discussions regarding patient cases, technical checks to inspect prevention and treatment plans, and performance appraisals. In the control areas, no service teams were assembled and current routine health services continued as usual. Patients in the intervention areas received two-hour lectures every two months, which focused on prevention and self-management strategies, nutrition and physical activity, health seeking behavior, and psychological counselling. They also received periodical follow-up interviews, an annual physical examination, and special medical services (including helping patients with medical treatment, transfer treatment, return visit, and clinical care). Patients in the control areas received routine services as usual. The intervention is explained more in detail in the study protocol [16]. This study followed the Consolidated Standards of Reporting Trials (CONSORT) Guidelines (Supplemental file). 

### 2.2. Study Population

In this study, we focused on the impact of services for patients in Jingjiang county. Patients were included in the study if they had been diagnosed with type 2 diabetes mellitus at county-level hospitals or if they satisfied the diagnostic criteria of the Chinese guidelines on the prevention and treatment of type 2 diabetes mellitus [17]. The inclusion criteria were as follows: being aged 35–75 years old, having lived in Jingjiang county for more than two years with no records of moving within the last year, having their own records in the chronic disease management information system of the township health center or village clinic, having accepted the chronic disease service provided by the township health center or village clinic, being willing to participate in the project, and having preferable compliance, cognition, and receptivity. Patients with serious diabetes-related complications, or those who had been diagnosed with any other serious disease, were pregnant, or had psychiatric disorders were excluded.

### 2.3. Data Collection

A questionnaire with 77 questions addressing diabetes treatment and knowledge about the disease, health-seeking behavior, continuity of and compliance to medical service, self-efficiency, satisfaction with care, medical treatment, transfer treatment, in-hospital treatment, questions on socioeconomic status, and HRQoL was provided to the study participants (intervention as well as control group) at baseline and at two follow-up data collections. In addition, the FBG levels were measured at baseline and follow-ups. Baseline data were collected in November 2015, and the follow-up data were collected in October 2016 and July 2017. For the present study, we used data collected at baseline and at the one-year follow-up, and analyzed the FBG levels, the HRQoL, and sociodemographic characteristics.

Questions on the patients’ sociodemographic characteristics concerned their age, sex, marital status, level of education, and occupation type. FBG level was measured in mmol/L from a venous blood sample. HRQoL was measured by the EQ-5D-3L questionnaire, which is a validated generic instrument used in clinical settings for general population and for economic evaluation [18]. The instrument consists of two parts: a descriptive system, and a vertical visual analogue scale (EQ VAS) [18]. In the descriptive system, respondents can report their health on five dimensions (mobility, self-care, usual activities, pain/discomfort, and anxiety/depression) and three levels of severity (no problem, some problems, or severe problems) [18]. By this classification, there are 243 possible unique health states that can be converted into an EQ-5D-3L index by adapting a value set, where dead = 0 and full health = 1. On the EQ VAS, the respondents can rate their overall health between best imaginable health state (100) and worst imaginable health state (0) [18]. The EQ-5D-3L index in this study was calculated by adopting the national Chinese EQ VAS value set developed by Sun et al. [19], where the values were transformed to the 0–1 scale.

### 2.4. Statistical Analyses

Participants’ ages were divided into five age groups: 36–49 years, 50–59 years, 60–64 years, 65–69 years, and 70–75 years. Marital status was divided into married (married or cohabiting) and single (unmarried; divorced or separated; widowed). Participants with primary school or lower education were defined into the low-education group. Participants with higher than primary school (middle school; high school; junior college; and bachelor or higher) were classified into the high-education group. Occupation type was divided into “farming or house working” and “others”. 

Data were analyzed using SPSS version 22.0 (SPSS Inc., Chicago, IL, USA) [20] and Stata 11.0 (College Station, TX, USA) [21]. The main outcome measures were FBG levels and self-reported health as expressed in the five EQ-5D-3L dimensions, the EQ VAS score, and the EQ-5D-3L index. Patients who reported “some problems” or “severe problems”, were classified as having “any problems”. To test for differences in sociodemographic characteristics between patients in the intervention and control groups, Pearson’s χ^2^-test or Fisher’s Exact test was used. Age was tested by independent t-test for differences between the control and intervention groups. FBG levels and prevalence of reporting any problems in the EQ-5D-3L dimensions, the EQ VAS score, and the EQ-5D-3L index were analyzed with the Mann–Whitney U test and the Pearson’s χ^2^-test/Fisher’s Exact test.

To test whether there were differences in outcomes between the intervention and control group before and after the intervention regarding the FBG levels, EQ VAS score, and EQ-5D-3L index, the difference-in-difference model (DID) was calculated [22]. The DID model is used to estimate the effect of an intervention or treatment, by comparing the changes in outcomes over time between a population that is enrolled in an intervention group and a control group [22]. In this study, we adopted two DID models: a crude model without adjustment and a model adjusted for age, sex, marital status, educational level, and occupation type. All statistical tests were carried out at 5% significance level.

## 3. Results

### 3.1. Response Rate

At baseline data collection in 2015, 267 questionnaires were collected from intervention townships, and 284 were collected from control townships. We excluded 8.2% (22) of the questionnaires in the intervention group and 12.7% (36) in the control group according to the exclusion criteria. At follow-up in 2016, 35 participants in the intervention group and 35 participants in the control group were lost to the follow-up. The final sample at follow-up consisted of 423 questionnaires: 213 in the intervention group and 210 in the control group. Among those lost to follow-up, 48 (68.6%) were unable to come to the township health centers due to a rainstorm, and the remaining 20 participants could not participate for other reasons (including death, travel, and moving).

### 3.2. Sociodemographic Characteristics of Study Participants

Table 1 shows the sociodemographic characteristics of participants at baseline. The mean age was 62.8 years and 73.5% of the participants were female. Most participants (85.6%) were married, and in the low-education group (63.8%). A majority of the participants (74.5%) were farmers or working in the home. There was a statistically significant difference between the intervention and control group regarding age, education level, and occupation. Participants in the intervention group were older, a higher proportion were females, with a low educational level, and farming/house working, compared with the control group.

### 3.3. Stratified Analyses

Table 2, Table 3, and Supplemental Table S1 show the FBG level, prevalence of problems by EQ-5D-3L dimensions, EQ VAS score, and EQ-5D-3L index for the intervention group and control group at baseline and follow-up for the whole sample (Table 2), and stratified by sex (Table 2), marital status (Table 2), age group (Supplemental Table S1), educational level (Table 3), and by occupation type (Table 3).

In the whole sample analysis (Table 2), there were statistically significant decreases in FBG levels and in the prevalence of reporting any problems in the dimensions of mobility, usual activities, and anxiety/depression in the intervention group. The mean EQ VAS score and EQ-5D-3L index for participants in the intervention group increased significantly at follow-up. In the control group, participants’ FBG levels increased significantly at follow-up, while the prevalence of problems in the dimensions of mobility, usual activities, pain/discomfort, and anxiety/depression decreased significantly at follow-up. The EQ-5D-3L index increased significantly at follow-up. In order to compare and summarize changes in different EQ-5D-3L dimensions in the intervention and control groups, a Paretian classification [23] of health change was made in both groups. There was some indication of more change (for better and for worse) in the intervention group compared to the control group, but no significant differences (data not shown).

In the analysis stratified by sex (Table 2), the decrease in FBG levels was only statistically significant among female participants in the intervention group. Females in the intervention group had a similar pattern as that of the whole sample analysis in the intervention group for the prevalence of any problem in the EQ-5D-3L dimensions, except that the decrease in the anxiety/depression dimension was not statistically significant. Females in the control group had a significant increase in FBG levels, as well as a significant decrease in prevalence of reporting any problem in all dimensions except self-care. Males in the intervention group reported a statistically significant decrease in prevalence of problems in the dimensions of pain/discomfort and anxiety/depression. Their mean EQ VAS score increased significantly at follow-up.

Married participants (Table 2) in the intervention group had a significant decrease in FBG levels and in prevalence of reporting any problem in all dimensions except self-care. They also had a significant increase in the mean EQ VAS score and EQ-5D-3L index. Single participants in the intervention group had no significant changes in any outcome.

In the analysis stratified by age (Supplemental Table S1), there was no significant result for FBG levels in any of the age groups. For the intervention group, among participants aged 50–59 years, a significant decrease of problems was found in the pain/discomfort dimension, and a significant increase was found in the EQ-5D-3L index. For participants aged 60–64 years, there was a significant decrease of problems in the dimension of usual activities. For participants aged 65–69 years, problems decreased significantly in the mobility dimension.

Participants with lower education (Table 3) in the intervention group experienced a significant decrease in FBG levels. They also had a similar pattern as in the total sample analysis for the prevalence of reporting any problem in different EQ-5D-3L dimensions. Among the participants with higher education in the intervention group, there was no significant difference in FBG levels, but a significant decrease in the prevalence of problems in the dimension of pain/discomfort, and a significant increase of the EQ-5D-3L index.

In the analysis stratified by occupation type (Table 3), participants in the intervention group whose occupation was farming or house working had similar changes as those in the total sample analysis for FBG levels and prevalence of any problems in the EQ-5D-3L dimensions. For participants in other types of occupations in the intervention group, the prevalence of problems in the pain/discomfort dimension decreased significantly at follow-up, while the EQ-5D-3L index increased significantly.

### 3.4. Difference-in-Difference Analysis

A DID analysis was conducted for the FBG level, mean EQ VAS score, and EQ-5D-3L index in order to test whether there were differences between the intervention and control groups before and after the intervention (Table 4). 

In the crude model, no differences were detected in FBG levels between the intervention group and control group at baseline. At follow-up, the FBG levels in the intervention group were significantly lower than those in the control group. The mean EQ VAS score and the EQ-5D-3L index in the control group were higher than in the intervention group at baseline. The DID analysis showed that the FBG levels in the intervention group decreased more than in the control group (where it increased). For mean EQ VAS score and EQ-5D-3L index, the DID model showed no statistical difference between the intervention and the control group at follow-up.

The results of the DID model adjusting for age, sex, marital status, educational level, and occupation type were similar to the unadjusted model.

## 4. Discussion

The follow-up one year after this education-based intervention among type 2 diabetes mellitus patients in rural China showed a positive impact on the FBG levels and some aspects of HRQoL in the intervention group compared to the control group. The FBG levels decreased significantly in the intervention group whereas it increased in the control group in the overall analysis as well as in the DID analysis. There were also signs of a differential impact of the intervention. In the stratified analysis, the FBG levels and some aspects of HRQoL improved in the intervention group more among females, those who were married, those with low education, and those in farming or house working. Participants in the control group had a deterioration in the FBG levels and in the prevalence of reported problems in some EQ-5D-3L dimensions, but also improved in mean EQ VAS score and the EQ-5D-3L index. 

The FBG levels improved, but the impact of the intervention on HRQoL and the different dimensions of EQ-5D-3L was less systematic. The significant decrease in prevalence of reported problems in the dimensions of mobility and usual activities may be due to the increased information and knowledge about physical exercise, healthy diet, and the importance of proper drug use in the intervention group. Most problems were reported in the dimension of pain/discomfort both in the intervention and the control group, at baseline and follow-up. Wang et al. [24] reviewed HRQoL among type 2 diabetes mellitus patients in Jiangsu province by using data from the Chinese National Health Service Survey 2013. The prevalence of reported problems by different EQ-5D-3L dimensions was similar to the present study, except that we observed a higher prevalence of problems in the anxiety/depression dimension. The mean EQ VAS score increased in the intervention group for the whole sample analysis, which may indicate that the participants perceived that their health improved after having had more information about physical activity, diet, healthy lifestyle, and diabetes self-management. This is in accordance with Parkin et al. [25], suggesting that EQ VAS includes something more related to individual health than the specific EQ-5D-3L dimensions, which might be reflected in the improved EQ VAS score in this study.

Female participants had lower FBG levels after the intervention, and a decrease in the proportion reporting any problems with mobility and usual activities. Married participants had greater improvement of the FBG levels, a decrease in reporting any problems in all the dimensions compared to single participants, and significantly increased mean EQ VAS scores and and EQ-5D-3L index. Married people may have more support from their family members in managing disease and changing lifestyle [26]. Meanwhile, the low statistical power may be another factor explaining the insignificant changes among single participants. The lack of statistically significant findings in the specific age groups observed may have different explanations, including lack of statistical power. Younger participants showed a larger decrease in FBG levels, although the decrease was not statistically significant. In the intervention group, lowly educated participants had a greater improvement in the FBG levels than did highly educated participants. This is somewhat surprising, as interventions have tended to be more successful among persons with higher education [15]. Similarly, farmers and house workers had greater improvement in the FBG levels than did participants in other types of occupation. This might indicate that this intervention was better suited for persons with a low educational level than with a high educational level. It seems that the intervention was effective for lowly educated persons and farmers in rural China.

When comparing the intervention and control groups, it is surprising to see a decreased proportion of reporting problems in the EQ-5D-3L dimensions and improved mean EQ VAS scores and EQ-5D-3L index in both the control and intervention groups, as the control group received no intervention. Few studies have found consistent results regarding the impact on HRQoL of educational interventions in type 2 diabetes mellitus [27]. Our results might partly be explained by compositional differences between the intervention and the control group; participants in the control group were younger, and more of them were married and had a higher educational level, all of which are associated with better HRQoL [28,29,30]. However, there was a deterioration in the FBG levels in the control group. As no intervention was provided to the control group, the changes in HRQoL may be due to other factors, including observation effects, the so-called “Hawthorne effect”, meaning that participants may modify their behavior in response to their awareness of being observed [31]. Improvements in HRQoL may take more than one year, but at the same time the person is getting older and HRQoL deteriorates with age. Therefore, the effects are difficult to predict. In addition, EQ-5D-3L may not be sensitive enough to capture small improvements in specific dimensions. Using the EQ-5D-5L with five severity levels might be more sensitive to smaller changes and may reduce the ceiling effect [32].

The changes in blood glucose levels in this study are similar to those observed in previous studies [33,34,35]. A number of other recent intervention studies, similar to the present study, have been reported in Chinese scientific journals [36,37,38,39]. The blood glucose level measured by FBG [36,37,38,39], 2 h postprandial blood glucose (2h PBG) [36,39], and the level of glycated hemoglobin (HbA1c) [37,38,39] decreased after the education-based intervention in those studies in a similar manner to that observed in the present study for the FBG level. Moreover, satisfaction with care [36,38], patients’ knowledge with diabetes [36], health behavior [37], patient-reported quality of life [38], and self-efficacy [39] all improved in those studies. 

There are several limitations in this study. The intervention and control groups differed in composition, which may blur the effect of the intervention. They differed with respect to sociodemographic characteristics: participants in the intervention group were older, and to a higher proportion female, with a low education level, and with farming or house working types of work, compared to the control group. These factors are all associated with poorer health, as also indicated by the lower EQ VAS score in the intervention group compared to the control group at baseline. The improvement in HRQoL in the intervention group might therefore have been greater, had the composition of the intervention and control groups been more similar. In addition, only one follow-up data collection was analyzed. Last but not least, being in a real-life setting, the implementation of the intervention may have varied, as the local health authority took charge of the implementation at each site, and details of the implementation are lacking.

## 5. Conclusions

The intervention improved FBG levels and some aspects of HRQoL among participants. Furthermore, the intervention seemed to differentially benefit females, married persons, lowly educated persons, and those in farming or house working more than other groups. 

## Figures and Tables

**Table 1:** Sociodemographic characteristics of participants at baseline data collection.

	Intervention Group (*n* = 213)		Control Group (*n* = 210)			*p*
	%	*n*	%	*n*		
Mean age (SD)	63.6 year (6.89)		61.5 year (9.10)			0.008
Age group						
36–49	2.8	6	11.9	25		0.049
50–59	21.1	45	25.7	54		
60–64	27.2	58	16.7	35		
65–69	29.1	62	23.8	50		
70–75	19.7	42	21.9	46		
Sex						
Male	20.2	43		32.9	69	0.003
Female	79.8	170		67.1	141	
Marital status						
Single	15.6	33		12.9	27	0.426
Married	84.4	179		87.1	183	
Education level						
Low education	68.5	146		59.0	124	0.042
High education	31.5	67		41.0	86	
Occupation type						
Farming or house working	78.9	168		70.0	147	0.036
Non-farming or others	21.1	45		30.0	63	

**Table 2:** Fasting blood glucose (FBG) level (mmol/L, mean value), prevalence (%) of any problems by EQ-5D-3L dimensions, EQ visual analogue scale (VAS) score (mean), and EQ index value (mean) for the intervention group (*n* = 213) and control group (*n* = 210) at baseline (2015) and follow-up (2016) for the total group and stratified by sex and by marital status.

		Total (*n* = 423)			Male (*n* = 112)			Female (*n* = 311)			Single (*n* = 60)			Married (*n* = 362)		
		2015	2016	*p*	2015	2016	*p*	2015	2016	*p*	2015	2016	*p*	2015	2016	*p*
FBG level	Intervention	8.30	7.90	**0.009 ^†^**	8.28	8.00	0.554 ^†^	8.32	7.87	**0.011 ^†^**	8.82	8.19	0.096 ^†^	8.19	7.86	**0.045 ^†^**
	Control	7.91	8.69	**0.003 ^†^**	7.79	8.62	**0.023 ^†^**	7.97	8.73	**0.042 ^†^**	7.89	8.48	0.365 ^†^	7.91	8.72	**0.005 ^†^**
EQ-5D-3L dimension																
Mobility																
Any problem	Intervention	22.5	11.3	**0.002 ^‡^**	27.9	11.6	0.058 ^‡^	21.2	11.2	**0.012 ^‡^**	12.1	10.3	1.000 ^§^	24.6	11.4	**0.001 ^‡^**
	Control	19.0	7.6	**0.001 ^‡^**	14.7	5.8	**0.033 ^‡^**	19.9	8.5	**0.006 ^‡^**	37.0	14.8	0.062 ^‡^	16.4	6.6	**0.003 ^‡^**
Self-care																
Any problem	Intervention	1.9	3.8	0.241 ^‡^	2.3	2.3	1.000 ^§^	1.8	4.1	0.199 ^‡^	0.0	6.9	0.215 ^§^	2.2	3.3	0.751 ^§^
	Control	3.8	1.9	0.241 ^‡^	2.9	2.9	1.000 ^§^	4.3	1.4	0.282 ^§^	7.4	0.0	0.215 ^§^	3.3	2.2	0.521 ^‡^
Usual activities																
Any problem	Intervention	13.1	3.8	**<0.001 ^‡^**	14.0	2.3	0.110 ^§^	12.9	4.1	**0.004 ^‡^**	6.1	6.9	1.000 ^§^	14.5	3.3	**<0.001 ^‡^**
	Control	13.3	2.4	**<0.001 ^‡^**	8.7	2.9	0.274 ^§^	15.6	2.1	**<0.001 ^‡^**	33.3	0.0	**0.002 ^§^**	14.4	2.7	**0.003 ^‡^**
Pain/discomfort																
Any problem	Intervention	55.9	46.9	0.065 ^‡^	53.5	27.9	**0.016 ^‡^**	56.5	51.8	0.384 ^‡^	45.5	58.6	0.301 ^‡^	57.5	45.1	**0.018 ^‡^**
	Control	42.4	31.4	**0.020 ^‡^**	31.9	26.1	0.453 ^‡^	47.5	34.0	**0.021 ^‡^**	66.7	29.6	**0.006 ^‡^**	38.8	31.7	0.155 ^‡^
Anxiety/depression																
Any problem	Intervention	23.5	14.1	**0.013 ^‡^**	23.3	4.7	**0.013 ^‡^**	23.5	16.5	0.104 ^‡^	15.2	10.3	0.713 ^§^	25.1	14.7	**0.012 ^‡^**
	Control	15.7	8.6	**0.025 ^‡^**	11.6	5.8	0.227 ^‡^	17.7	9.9	0.058 ^‡^	25.9	7.4	0.142 ^§^	14.2	8.7	0.101 ^‡^
EQ VAS score	Intervention	74.80	77.20	**0.037 ^†^**	78.56	81.28	0.504 ^†^	73.85	76.21	0.051 ^†^	76.67	76.03	0.912 ^†^	74.37	77.42	0.020 ^†^
	Control	77.44	78.23	0.290 ^†^	78.90	80.20	0.664 ^†^	76.72	77.27	0.327 ^†^	70.96	74.63	0.246 ^†^	78.39	78.77	0.523 ^†^
EQ-5D-3L index	Intervention	0.870	0.900	**0.002 ^†^**	0.863	0.944	**0.002 ^†^**	0.866	0.893	**0.050 ^†^**	0.905	0.892	0.496 ^†^	0.858	0.905	**<0.001 ^†^**
	Control	0.894	0.932	**<0.001 ^†^**	0.921	0.945	0.120 ^†^	0.881	0.926	**0.001 ^†^**	0.816	0.934	**0.001 ^†^**	0.906	0.932	**0.014 ^†^**

†. Calculated by Mann–Whitney *U* test; ‡. Calculated by Pearson Chi-square test; §. Calculated by Fisher’s Exact test. Bold format: The p-value is less than 0.05.

**Table 3:** FBG level (mmol/L, mean value), prevalence (%) of any problems by EQ-5D-3L dimensions, EQ VAS score (mean), and EQ index value (mean) for the intervention group (*n* = 213) and control group (*n* = 210) at baseline (2015) and follow-up (2016) stratified by education level and by occupation type.

		Low Education (*n* = 270)			High Education (*n* = 153)			Farming/House Working (*n* = 315)			Other Types (*n* = 108)		
		2015	2016	*p*	2015	2016	*p*	2015	2016	*p*	2015	2016	*p*
FBG level	Intervention	8.4	8.0	**0.028 ^†^**	8.2	7.7	0.141 ^†^	8.4	8.0	**0.035 ^†^**	8.1	7.4	0.146 ^†^
	Control	7.8	8.9	**0.003 ^†^**	8.1	8.4	0.325 ^†^	7.9	8.6	0.086 ^†^	7.9	8.9	**0.009 ^†^**
EQ-5D-3L dimension													
Mobility													
Any problem	Intervention	26.7	13.4	**0.004 ^‡^**	13.4	6.3	0.169 ^‡^	24.4	13.0	**0.006 ^‡^**	15.6	2.8	0.070 ^§^
	Control	25.0	10.1	**0.002 ^‡^**	10.5	3.7	0.091 ^‡^	23.8	7.0	**<0.001 ^‡^**	7.9	8.6	0.883 ^‡^
Self-care													
Any problem	Intervention	1.4	4.7	0.173 ^§^	3.0	1.6	1.000 ^§^	1.8	4.0	0.338 ^§^	2.2	2.8	1.000 ^§^
	Control	6.5	1.6	0.056 ^§^	0.0	2.5	0.234 ^§^	5.4	0.0	**0.010 ^§^**	0.0	4.3	0.148 ^§^
Usual activities													
Any problem	Intervention	16.4	4.7	**0.001 ^‡^**	6.0	1.6	0.366 ^§^	14.3	4.0	**0.001 ^‡^**	8.9	2.8	0.375 ^§^
	Control	16.9	2.3	**<0.001 ^‡^**	8.1	2.5	0.170 ^§^	17.7	0.0	**<0.001 ^‡^**	3.2	5.4	0.702 ^§^
Pain/discomfort													
Any problem	Intervention	60.3	56.4	0.497 ^‡^	46.3	25.0	**0.011 ^‡^**	58.3	52.0	0.236 ^‡^	46.7	22.2	0.023 ^‡^
	Control	49.2	34.1	**0.015 ^‡^**	32.6	27.2	0.447 ^‡^	49.0	33.9	**0.014 ^‡^**	27.0	28.0	0.894 ^‡^
Anxiety/depression													
Any problem	Intervention	28.1	17.4	**0.029 ^‡^**	13.4	6.3	0.169 ^‡^	26.8	16.4	**0.019 ^‡^**	11.1	2.8	0.219 ^§^
	Control	19.4	11.6	0.089 ^‡^	10.5	3.7	0.091 ^‡^	18.4	11.3	0.115 ^‡^	9.5	5.4	0.353 ^§^
EQ VAS score	Intervention	73.3	76.0	**0.038 ^†^**	78.0	80.0	0.367 ^†^	73.3	75.7	0.058 ^†^	80.4	84.9	0.062 ^†^
	Control	76.3	76.1	0.760 ^†^	79.0	81.7	0.157 ^†^	76.1	77.3	0.284 ^†^	80.6	79.3	0.815 ^†^
EQ-5D-3L index value	Intervention	0.849	0.883	**0.022 ^†^**	0.902	0.949	**0.010 ^†^**	0.856	0.891	**0.010 ^†^**	0.901	0.961	**0.008 ^†^**
	Control	0.871	0.921	**0.001 ^†^**	0.927	0.950	0.104 ^†^	0.874	0.932	**<0.001 ^†^**	0.940	0.933	0.718 ^†^

†. Calculated by Mann–Whitney U test; ‡. Calculated by Pearson Chi-square test; §. Calculated by Fisher’s Exact test. Bold format: The p-value is less than 0.05.

**Table 4:** FBG level (mmol/L, mean value), prevalence (%) of any problems by EQ-5D-3L dimensions, EQ VAS score (mean), and EQ index value (mean) for the intervention group (*n* = 213) and control group (*n* = 210) at baseline (2015) and follow-up (2016) stratified by education level and by occupation type.

	2015				2016				Crude		Adjusted	
	Control	Interven-tion	Difference	*p*	Control	Interven-tion	Difference	*p*	DID	*p*	DID	*p*
FBG	7.91	8.31	0.40	0.084	8.69	7.90	−0.79	0.001	−1.19	<0.001	−1.22	<0.001
EQ VAS score	77.4	74.8	−2.6	0.039	78.2	77.2	−1.0	0.423	1.6	0.365	2.1	0.167
EQ-5D-3L index value	0.89	0.86	−0.03	0.014	0.93	0.90	−0.03	0.012	0.00	0.970	0.00	0.849

## Supplementary Materials

The following are available online at https://www.mdpi.com/1660-4601/16/15/2676/s1: Supplemental Table S1. FBG level (mmol/L, mean value), prevalence (%) of any problems by EQ-5D-3L dimensions, EQ VAS score (mean), and EQ index value (mean) for the intervention group (*n* = 213) and control group (*n* = 210) at baseline (2015) and follow-up (2016) stratified by age group. Supplemental file: CONSORT 2010 checklist of information to include when reporting a randomized trial.
